# Entrainment of marginally stable excitation waves by spatially extended sub-threshold periodic forcing

**DOI:** 10.1186/1753-4631-5-8

**Published:** 2011-09-25

**Authors:** Joseph M Starobin, Vivek Varadarajan

**Affiliations:** 1 University of North Carolina at Greensboro, North Carolina, 27402, USA

## Abstract

We introduce a novel approach of stabilizing the dynamics of excitation waves by spatially extended sub-threshold periodic forcing. Entrainment of unstable primary waves has been studied numerically for different amplitudes and frequencies of additional sub-threshold stimuli. We determined entrainment regimes under which excitation blocks were transformed into consistent 1:1 responses. These responses were spatially homogeneous and synchronized in the entire excitable medium. Compared to primary pulses, pulses entrained by secondary stimulations were stable at considerably shorter periods which decreased at higher amplitudes and greater number of secondary stimuli. Our results suggest a practical methodology for stabilization of excitation in reaction-diffusion media such as nerve tissue with regions of reduced excitability.

## Background

Dynamics of excitation waves in reaction-diffusion media can be altered by spatio-temporal periodic forcing. Additional (secondary) periodic stimulations superimposed on primary forcing may alter the primary excitation waves and entrain (lock) them to the period of secondary stimuli. Locking of primary waves to the period of secondary stimulations occurs at particular values of forcing periods and amplitudes. This resonant shift is characterized by Arnold tongues which determine the margins of different types of *M *: *N *(*M *≥ *N*, *N *≥1) locking responses as a function of amplitude and frequency of external forcing [1,2].

It was found that the phenomenon of locking manifests itself in different ways depending on the spatio-temporal complexity of the particular reaction-diffusion system. For example, under the periodic forcing, spatially uniform two-dimensional BZ reaction oscillations were transformed into standing wave type labyrinths of complex geometry [1]. It was also demonstrated that one-dimensional Turing patterns can be modulated by spatio-temporal forcing in the form of a travelling wave [3].

A similar type of resonant behaviour of Turing patterns was observed in experiments with photosensitive chemical reactions [4]. It was also shown that the presence of large amplitude periodic forcing in a one-dimensional bistable reaction-diffusion medium resulted in bifurcating of an originally stable wavefront into two counter propagating wavefronts [5]. In contrast, modulating the intensity and frequency of the periodic forcing controlled the trajectory and rotational frequency of two-dimensional spiral waves in the Oregonator model [6].

Additional periodic forcing can be also applied for locking of primary waves in some biological excitable media. A typical example of practical realization of such resonant dynamics is a post-traumatic adjustment of excitation in nerves with impaired excitability. It was shown that impaired excitation may be restored by applying additional functional electrical stimulation using implantable [7] or body surface stimulation electrodes [8-10]. This method has been confirmed as an effective tool for restoration of movement of paralyzed muscles in individuals with a variety of neurological impairments [11].

Usually, after severe neuromuscular injuries nerve conductivity is significantly reduced, which in turn, prevents the passage of excitation waves through neuromuscular transmitters. Under these circumstances propagation of excitation pulses is marginally stable and implementation of functional electrical stimulation necessitates a significant increase of frequencies and amplitudes of additional electrical stimuli. The latest, instead of stabilization of propagation, can facilitate conduction blocks and may completely disrupt the process of training paralyzed muscles.

To address this deficiency we investigate a new approach of entrainment of marginally stable excitation waves by spatially extended low amplitude sub-threshold forcing. We demonstrate that such sub-threshold forcing can transform excitation blocks to stable 1:1 responses synchronized in the entire excitable medium.

## Methods

Dynamics of conduction in nerves is simulated by Fitzhugh-Nagumo type reaction-diffusion equations with just two fundamental excitation and post-excitation recovery variables [12,13]. Unlike our recent work where we studied the dynamics initiated by a single excitation source [14], the adjusted model is set up to reflect the interference of several sources which deliver multiple stimuli of different amplitudes. In particular, the main source delivered localized over-threshold stimuli applied at the beginning of the cable and a set of additional sources originated secondary sub-threshold forcing which was extended throughout the entire cable (Eqs. 1).

(1) $$\begin{array}{llll} \frac{\partial u}{\partial t} & =\frac{\partial^{2}u}{\partial x^{2}}-i\left(u,v\right)\;+P\left(x,t\right)\;+\overset{n}{\underset{i=1}{\sum }}S\left(x-x_{i},t\right)\; & & \;\\ & \;i\left(u,v\right)=\left\{\begin{array}{cc} \lambda u, & u<v\\ u-1, & u\geq v \end{array}\right)\; & & \;\\ & \;\frac{\partial v}{\partial t}=\varepsilon \left(\zeta u+v_{r}-v\right)\; & & \;\\ & \; & \end{array}$$

Here *u *and *v *are dimensionless excitation and recovery functions, respectively. ε is a small parameter and *v_r _*is the excitation threshold. λ and ζ control the rates of changes of excitation and recovery functions, respectively.

The system of Eqs. 1 was solved numerically in a one-dimensional cable of finite length using a second order explicit difference scheme with zero flux boundary conditions [15]. Spatial, Δ*x*, and temporal, Δ*t*, steps used in the numerical integration were equal to 0.23 and 0.0072, respectively. The cable length, *L*, was equal to 150Δ*x*. Primary forcing, *P *(*x*, *t*), was a train of rectangular pulses with duration 100Δ*t*, an over-threshold amplitude *A*_0 _and period *T*_0_. Primary stimuli were applied near the left end of the cable between *x *= 2Δ*x *and *x *= 15Δ*x*. Secondary forcing {*S *(*x - x_i_*, *t*), *i *= 1,2,..., *n*} also a rectangular pulse train with duration 100 Δ*t*, was delivered at *n *equidistant locations between *x *= 40Δ*x *and *x *= 140Δ*x*. The secondary stimuli were simultaneously activated after the wavefront which resulted from the first primary stimulation arrived at the end of the cable. The amplitude and period of secondary stimulations were equal to *A *and *T*, respectively.

**Figure 1:**
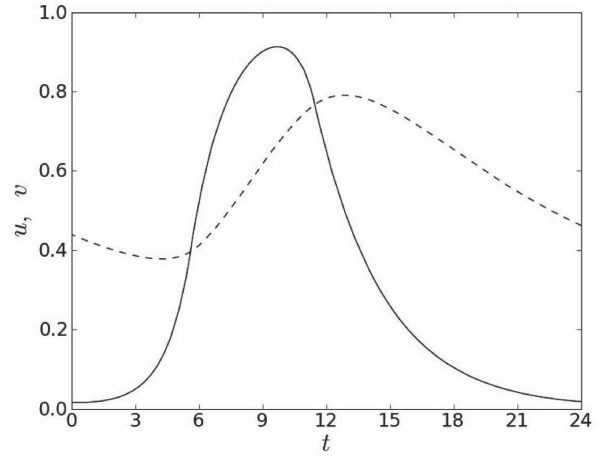
**Excitation, *u*, (solid line) and recovery, *v*, (dashed line) variables at *x *= *L */2 for the short steady state pulse**. Left and right intersections between *u *and *v *mark the beginning and the end of the pulse, respectively.

Unless mentioned otherwise, values of parameters in all computations were *ε *= 0.1, λ = 0.4, *ζ *= 1.2, *A*_0 _= 1.4, *α* = 0.31, *β *= 0.0025 and *n *= 6. As in [14] we used a simplified primary rate dependent excitation threshold given by linear equation *v_r _*= *α *- *βT*_0_, *α* > 0, *β *> 0. The duration of a pulse, *T_h_*, was measured as the time interval between consecutive intersections of *u *and *v *near their rest and excited states, respectively (Figure 1). The steady state value of *T_h _*was computed after 80 primary stimulation periods.

## Results

**Figure 2:**
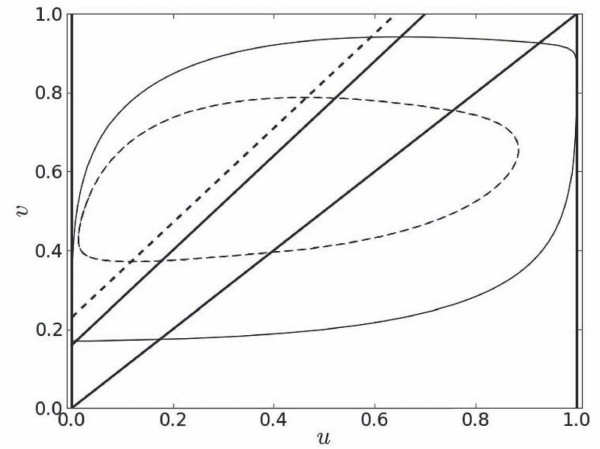
**Phase portraits of the system at *x *= *L/2 *for *T*_0 _= *T_end _*= 30 (dashed contour) and *T*_0 _= 60 (solid contour)**. Nullcline of *u *is given by thick N-shaped line. Dashed and solid lines with intercepts at *v_r _*= 0.23 and *v_r _*= 0.16 are nullclines of *v *for *T*_0 _= 30 and *T*_0 _= 60, respectively.

In the absence of secondary stimulation (*A *= 0), at long periods *T*_0 _the variable *v *has enough time to reach its steady state *v_r _*before the next stimulus is applied. However, as *T*_0 _is reduced to a critical limit, *T_end_*, the system does not respond to every stimulus and exhibits unstable *M *: *N *(*M *>*N *) excitation blocks which occur due to incomplete recovery of control variable *v*. Figure 2 compares phase portraits of the system at two values of *T*_0_. At *T*_0 _= 60, *u *closely follows its nullcline and *v *almost completely recovers to its steady state value of 0.16 as depicted by the intersection of the *u *- *v *nullclines. However, at *T*_0 _= *T_end _*= 30, deviations of *u *and *v *from their nullclines are quite significant, thereby *v *recovers to a value which is much higher than the corresponding threshold of 0.23.

**Figure 3:**
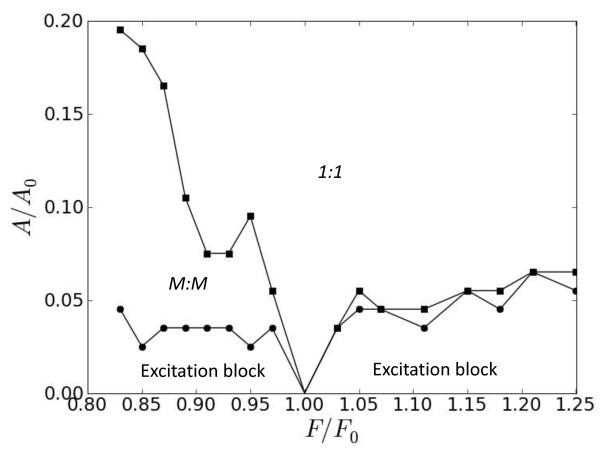
**Locking margins for different frequencies and amplitudes of secondary stimuli for *T*_0 _= 30, *A*_0 _= 1.4, $x=\frac{L}{2}$**.

Analysis of pulse duration *T_h _*at the critical primary period *T_end _*= 30, as a function of secondary frequency *F *= *T *^-1 ^and forcing amplitude *A *≥ 0 revealed a variety of entrainment regimes (Figure 3). We found that the system did not respond to secondary stimuli at amplitudes which were smaller than critical values depicted by the curve with circular markers. For the amplitudes above this curve and frequencies smaller than *F*_0 _we observed intermediate *M *: *M *responses with *M *greater than one.

For even greater amplitudes, above the upper curve with square markers, the system locked to secondary stimuli with consistent 1:1 responses. It should be noticed that such locking occurred over a wide range of secondary frequencies, and that secondary amplitudes were five times smaller than the amplitudes of primary stimulations. We also observed that for frequencies greater than *F*_0 _the entrainment of blocked excitation occurred without intermediate *M:M *responses (Figure 3).

**Figure 4:**
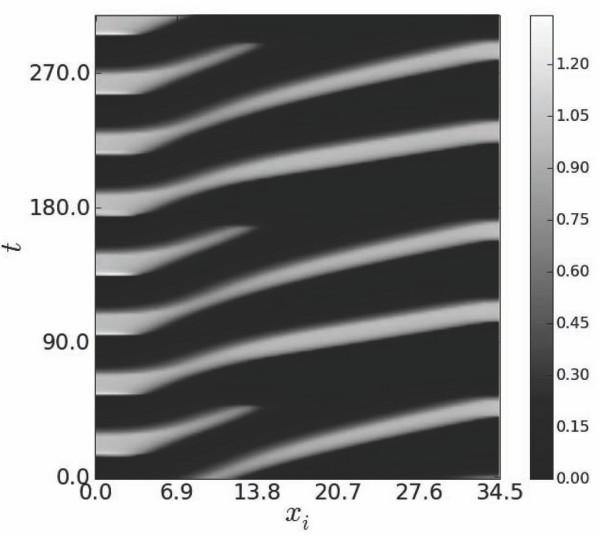
**Gray-scale patterns in the spatio-temporal evolution of *u***. Corresponding scale bar is shown on the right.

**Figure 5:**
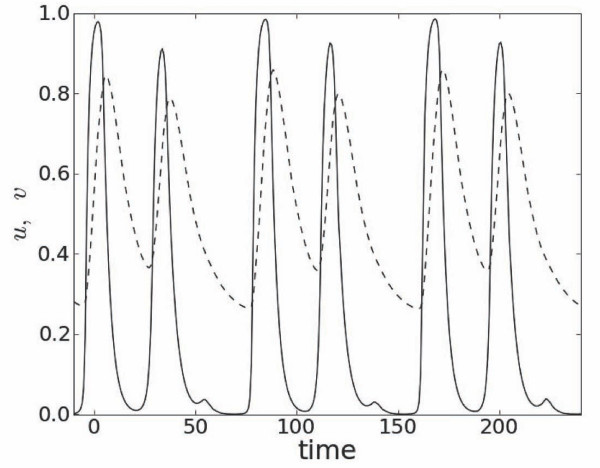
**Dynamics of *u *(solid line) and *v *(dashed line) for three consecutive cycles at *T*_0 _= 28, *A*_0 _= 0, $x=\frac{L}{2}$**.

**Figure 6:**
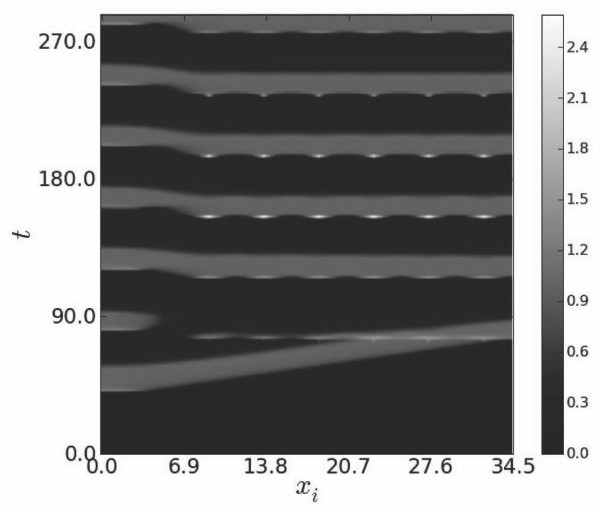
**Gray-scale bands depicting synchronization of *u *for *T*_0 _= *T *= 28 and *A *= 0.1*A*_0_**. Corresponding scale bar is shown on the right.

Spatio-temporal contours of *u *shown in Figure 4 demonstrate expected unstable responses to primary stimulation at *T*_0 _<*T_end_*. Temporal dynamics of *u *and *v*, as well as spatio-temporal contours of *u*, show 3:2 excitation blocks (Figure 4,5). However, in the presence of secondary stimulations such unstable responses can be entrained and stabilized by secondary driving even at *T*_0 _<*T_end_*. Indeed, Figure 6 demonstrates that 3:2 blocks can be transformed into stable 1:1 responses which evolve homogeneously in the entire cable except for short segments located near the site of primary stimulation.

**Figure 7:**
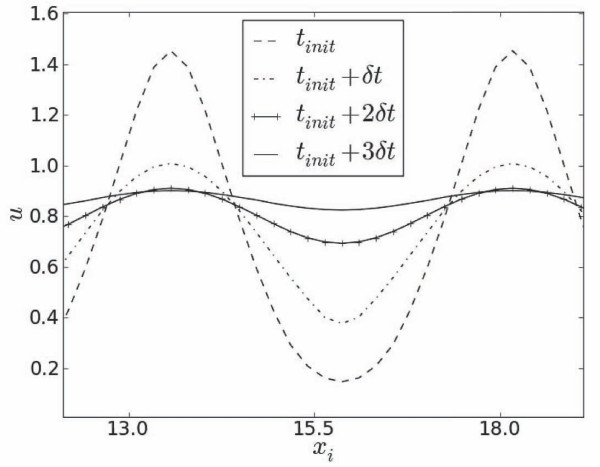
**Temporal evolution of *u *near the bottom of the second band as depicted in Fig. 6**. Profiles are shown for three equidistant moments of time, starting at *t_init _*= 136, *δt *= 0.36.

Formation of these fully synchronized responses are preceded by very short (~ 0.02*T*_0_) transient periods during which standing wave type oscillations of *u *rapidly saturate at constant excitation levels (Figure 7).

**Figure 8:**
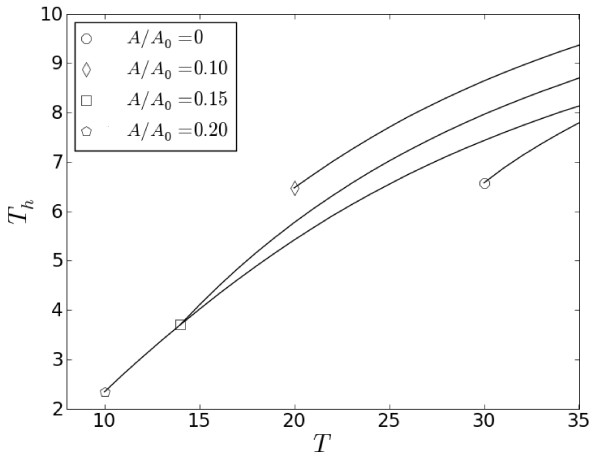
**Dependence of *T_h _*on *T *shown for different amplitudes of secondary stimuli at *T*_0 _*= T***. Ends of the curves at *T_end _*for different amplitude ratios are indicated by corresponding markers.

**Figure 9:**
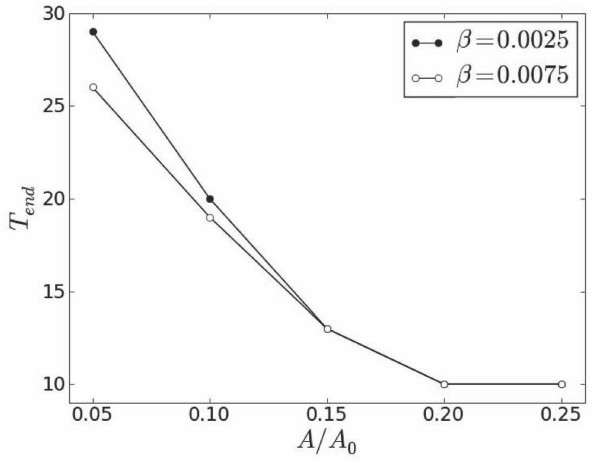
**Dependence of *T_end _*on $\frac{A}{A_{0}}$ for two values of *β *at $x=\frac{L}{2}$**.

When compared with primary forcing alone (*A *= 0), secondary sub-threshold stimuli facilitate development of stable pulses shorter than those at *T_end _*= 30. Figure 8 shows that secondary stimuli of higher amplitudes sustain shorter entrained pulses at progressively smaller stimulation periods. The trend can be slightly augmented for higher coefficients *β *when *T_end _*decreases as shown in Figure 9.

**Figure 10:**
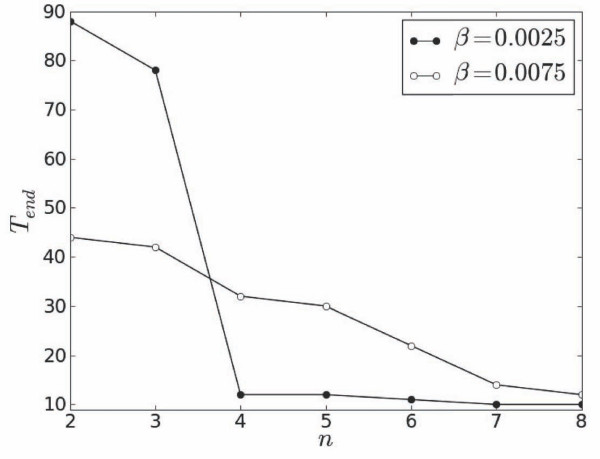
**Dependencies of *T_end _*on *n *for different *β*, *α *= 0.67 and $x=\frac{L}{2}$**.

Stabilization of the system due to secondary driving can also be achieved using a greater number of secondary stimulation sources. Correspondingly, Figure 10 demonstrates that an initial two-fold increase of the number of secondary sources from 2 to 4 extends the region of stability towards shorter values of *T_end_*. Further increase of the number of sources saturates these changes at progressively shorter values of *T_end _*for smaller coefficients *β*.

## Conclusions

In summary, we demonstrated that additional sub-threshold driving stimuli can entrain otherwise unstable primary reaction-diffusion waves and transform *M *: *N *excitation blocks into stable 1:1 spatially homogeneous responses synchronized in the entire cable. Compared to pulses resulting from primary forcing alone, pulses entrained by secondary stimulations were stable at considerably shorter periods. These periods decreased at higher amplitudes and greater number of secondary stimuli. We also found that locking to stable 1:1 responses occurred over a wide range of secondary frequencies. In addition, the sub-threshold secondary amplitudes were a factor of five smaller than the amplitudes of primary stimuli. Our results outline the possibility of entrainment of reaction-diffusion waves by sub-threshold additional driving and may be applied for stabilization of excitation in nerves with regions of impaired excitability [16].

## Competing interests

The authors declare that they have no competing interests.

## Authors' contributions

JS conceived, developed and designed the theory and numerical experiments. VV performed the numerical simulations and has been involved in drafting and revising the manuscript. Both authors read and approved the final manuscript.
